# Patient identified with interrupted aortic arch and 22q11.2 deletion syndrome in late pregnancy: a case report

**DOI:** 10.1177/03000605241306905

**Published:** 2024-12-29

**Authors:** Kejun Pu, Mengya Yang, Meng Xia, Shuxin He, Hongbi Song, Jing Wu

**Affiliations:** 1 The Second Clinical Medical College, 326770 Guizhou University of Traditional Chinese Medicine, Guiyang, Guizhou Province, China; 2 Department of Obstetrics, Guizhou Provincial People’s Hospital, Guiyang, Guizhou Province, China; 3 Intensive Care Unit, Guizhou Provincial People’s Hospital, Guiyang, Guizhou Province, China

**Keywords:** Interrupted aortic arch, atypical adult, 22q11.2 deletion syndrome, pregnancy, case report, whole-exome sequencing

## Abstract

Interrupted aortic arch (IAA) is an uncommon congenital heart malformation that can be fatal with no surgical intervention. Most patients have IAA discovered as newborns because of cardiac symptoms, and atypical adult patients are relatively rare. We report a case of atypical adult IAA in a woman in her 20s. Her first pregnancy and childbirth did not show any obvious abnormalities. Although this patient did not have any major clinical manifestations, she developed tachycardia during her second pregnancy and was diagnosed with IAA by echocardiography. The etiology of IAA was chromosome 22q11.2 deletion syndrome based on the results of whole-exome sequencing. After interdisciplinary diagnosis and treatment to reduce the pulmonary artery pressure and cardiac burden, the patient and her newborn had a favorable prognosis. This case highlights the importance of not overlooking cardiovascular concerns in patients who develop symptoms in pregnancy. Echocardiography can detect IAA, and genetic testing may assist in an etiological diagnosis, which improves the maternal and fetal prognosis.

## Background

Interrupted aortic arch (IAA) is an uncommon congenital heart malformation. The incidence of IAA is 1/30,000, accounting for 1% of congenital heart malformations.^1,2^ A total of 90% of patients with IAA die of severe heart failure when they are newborns if there is no surgical repair, and a small number of patients survive to adulthood.^3^ The definition of IAA is continuous interruption of the aortic arch, usually manifesting as an interruption of the connection between the ascending aorta and the descending aorta.^1–3^ The common cardiac symptoms associated with this malformation include severe pulmonary hypertension, ventricular septal defect, heart failure, and severe cyanosis. IAA is easily found in newborns because of pneumonia, heart failure, severe cyanosis, and other symptoms. Without surgical intervention, the survival rate of IAA is low.^4^ If echocardiography can detect abnormalities in the fetal aortic arch and surgical intervention can be performed in a timely and successful manner after birth, the prognosis is usually good.^2,3^ Therefore, IAA is less likely to be diagnosed in adulthood without surgical repair, and atypical adults are rare.

We report a case of atypical adult IAA. The findings in this case confirm that even atypical adults may have IAA, and this malformation can have an occult nature in surviving adult patients. Patients with IAA are often accompanied by congenital genetic defects, which need to be identified by genetic tests. Especially in pregnant patients, early detection and treatment of IAA may improve the maternal and fetal prognosis.

## Case presentation

A woman in her early 20s with natural conception was admitted to the hospital at 40^+5^ weeks of pregnancy. She graduated from junior high school and did not show any specific learning difficulties during her school years. Her parents and siblings did not have similar disorders. She had no history of adverse pregnancies and had given birth to a healthy girl 2 years previously. The patient did not develop scoliosis or renal malformations, and her thyroid examination and immune function were normal.

**Figure 1.**
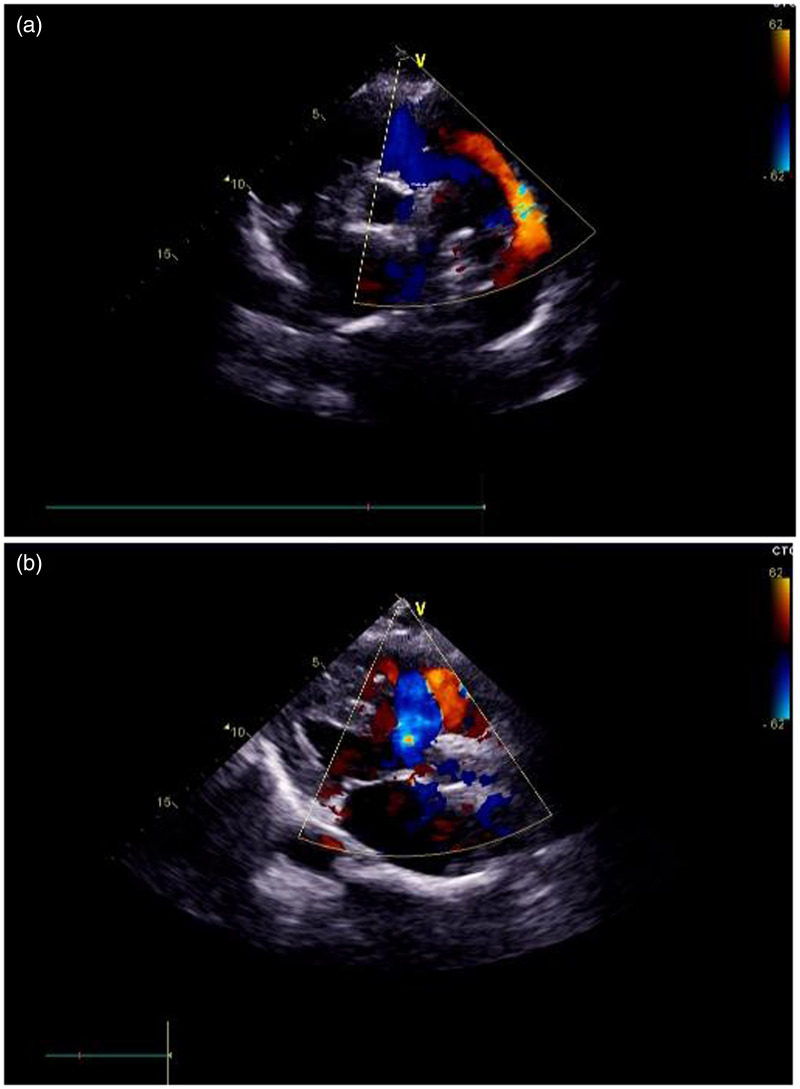
Echocardiographic images. (a) Enlargement of the left atrium and left ventricle and an abnormal conduit connecting the left pulmonary artery and descending aorta can be seen. (b) This image shows the morphology of the aortic valve and pulmonary valve, the morphology of the mitral valve and tricuspid valve, and interruption of echo in the interventricular septum.

Her second pregnancy was discovered at 8 weeks of gestation. At 12 weeks of gestation, the fetal nuchal translucency was normal. She did not choose to have early Down syndrome screening, which suggested a low risk in the second trimester. At 22 weeks of gestation, anatomical ultrasound examinations were normal. At 38 weeks of gestation, the patient developed palpitation and chest tightness. She went to the clinic for a check-up, and an electrocardiogram indicated tachycardia, an incomplete right bundle branch block, and high left ventricular voltage. The patient was transferred to our hospital at 40^+5^ weeks of gestation because of limited local medical services. After admission, she manifested palpitation, chest tightness, shortness of breath, and cyanosis of the lips. The patient did not show differential cyanosis in clinical manifestations. The patient’s vital signs were as follows: temperature, 36.5°C; heart rate, 100 beats/minute; and blood pressure, 102/57 mmHg. A physical examination suggested a grade 3/6 systolic murmur in the pulmonary valve area, a grade 4/6 systolic murmur in the second auscultation area of the aortic valve, palpable tremors, enlargement of the heart boundary to the lower left, and apical pulsation. Echocardiography showed congenital heart disease, which comprised ventricular septal defect (perimembranous type with a left to right bidirectional shunt), patent ductus arteriosus (continuous bidirectional shunt at the level of great vessel), accelerated anterograde flow in the left pulmonary artery (a possible interruption of the aortic arch), mild to moderate mitral regurgitation, mild tricuspid regurgitation, and severe pulmonary arterial hypertension (estimated to be 148 mmHg) (Figure 1).

According to the clinical manifestations and examination results, the patient was diagnosed with pregnancy complicated by IAA (possibly type B), ventricular septal defect (perimembranous type), severe pulmonary hypertension, and cardiac function level of New York Heart Association class II to III. When the patient was diagnosed with IAA, we immediately suggested a comprehensive cardiac computed tomography angiography examination to confirm the diagnosis of IAA and further assess the aortic arch anatomy and any associated vascular abnormalities. However, she refused to undergo this examination.

The most common genetic finding associated with IAA is 22q11.2 deletion syndrome (22q11.2DS), and a chromosomal microarray analysis is the standard first-line test when this is suspected. In our case, the patient did not have any other symptoms of 22q11.2DS, and none of her family members had this syndrome. Therefore, we could not rule out the possibility of other genetic diseases besides 22q11.2DS. Consequently, we performed whole-exome sequencing (WES) rather than a chromosomal microarray to determine the etiology of IAA. We found that there was a copy number deletion of approximately 2.35 Mb in the long arm of chromosome 22, which was preliminarily determined as a pathogenic mutation. This patient was ultimately diagnosed with 22q11.2DS with IAA as the main clinical manifestation.

The patient had an emergency cesarean section performed under epidural anesthesia after being administered therapies, such as cardiac strengthening and reducing pulmonary artery pressure. The surgical process went smoothly with a bleeding volume of approximately 400 mL. Her vital signs remained stable during the operation. Her vital signs remained stable during the operation and a female neonate weighing 2575 g was delivered. The Apgar score was 9 points at 1 minute and 10 points at 5 minutes. The newborn was transferred to the neonatal intensive care unit because of her high risk. An examination of the newborn showed no signs of scoliosis or renal malformation. The creatine kinase isoenzyme level was 125.80 U/L and the indirect bilirubin level was 29.5 µmol/L. An electrocardiogram showed a heart rate of 150 beats/minute. Treatment of reduced myocardial enzymes and blue light irradiation for 3 days resulted in a decrease in the neonatal creatine kinase isoenzyme level to 35.70 U/L and the indirect bilirubin level returned to normal. On the 5th day, the neonate was discharged from the hospital.

The patient was immediately transferred to the intensive care unit after the cesarean section and monitored for vital signs. On the basis that echocardiography still showed severe pulmonary hypertension after surgery (66 mmHg), she continued to receive diuretics, treprostinil, and oxygen therapy to reduce the pulmonary hypertension. However, on the 5th day after the operation, the patient suddenly showed a decrease in blood pressure, accompanied by cyanosis of the lips and shortness of breath. A physical examination showed the following abnormalities: heart rate, 98 bears/minute; blood pressure, 77/36 mmHg; oxygen saturation, 80%; and a coarse full-systolic murmur of grade 3/6 between the left 3/4 costal margin of the sternum. In consideration of the possibility of a pulmonary hypertension crisis, we immediately administered an increased dosage of treprostinil, high-flow oxygen therapy, and natriuretic peptide drugs to alleviate pulmonary arterial pressure and reduce the right heart load. After the above-mentioned treatment measures, the blood pressure and oxygenation of the patient gradually stabilized and her vital signs returned to normal. She was discharged on the 18th day. The patient took oral ambrisentan tablets as sequential treatment after discharge. In a follow-up visit, we attempted to contact the patient several times, and suggested that she have corrective cardiac surgery at an elective time. However, she still refused to undergo cardiac correction surgery. We advised her that 22q11.2DS has a high heritability, and her offspring and parents should undergo genetic testing. However, the patient did not follow our advice.

The reporting of this study conforms to the CARE guidelines.^5^ We did not obtain signed consent to publish from the patient. We have de-identified all of the patient’s details. We obtained consent from the patient for treatment. This case report was approved by the ethics review committee of Guizhou Provincial People’s Hospital (No. 2018040).

## Discussion

IAA is a rare congenital heart malformation and often associated with severe congenital aortic malformations. In the adult cases of IAA reported in recent years, most were men and accompanied by hypertension, shortness of breath, and pain from the chest and back.^6–9^ In contrast, our patient was a young pregnant woman who had no detectable cardiac abnormalities in her first pregnancy. During this pregnancy, there was no hypertension, chest or back pain, chest tightness, shortness of breath, or other symptoms. She developed tachycardia during her second pregnancy and was diagnosed with IAA by echocardiography. This atypical adult is relatively rare among the reported cases of IAA.

IAA is defined as an abnormal separation of the aortic arch and the descending aorta, and is often divided into three types depending on the loss of vascular connection. Type A presents with disconnection of the aortic arch to the descending aorta at the distal end of the left subclavian segment. This type does not show aortic stenosis and has an incidence of approximately 40%. Patients with type A IAA rarely have 22q11.2DS. Type B is caused by interruption of the aortic arch between the left common carotid artery and the left subclavian artery. The pathological and physiological changes of type B are impaired blood flow from the left ventricle and loss of the interventricular septum, combined with a reduction in blood flowing to the aortic arch. In recent years, 20% of type B IAA has been reported in 22q11.2DS.^2,3^ In patients with type C IAA, the ascending aorta forms the brachiocephalic trunk upward. The left common carotid artery and left subclavian artery are connected to the descending aorta because of no arterial arch structure. Type C is the smallest proportion of IAA and is rarely found in 22q11.2DS.^10^ In our case, echocardiography showed an abnormal conduit with an inner diameter of approximately 15 mm between the left pulmonary artery and the descending aorta. The brachiocephalic artery and the left subclavian artery originated from the aortic arch, while the other end of the aortic arch was a blind end. The abnormality of the aortic arch indicated that the presence of horizontal shunting of large blood vessels and the other end of the aortic arch was a blind end (possibly type B). Based on the clinical manifestations, physical examination, and results of echocardiography, the patient was diagnosed with pregnancy complicated by IAA (possibly type B), and the New York Heart Association class was II to III.

Some researchers have found that the pathogenesis of IAA is related to deletion of the 22q11.2 gene locus.^11^ However, some clinical features of 22q11.2DS may indicate the presence of IAA. Angelo DiGeorge first described 22q11.2DS in 1965 in a genetic analysis of thymic and parathyroid tissue.^12^ The incidence of 22q11.2DS is 1/2148, and the clinical manifestations are individual and diverse. The main characteristics reported include congenital heart disease, special facies, congenital palate abnormalities, immunodeficiency, learning difficulties, and mental disorders.^12,13^ Approximately 80% of patients with 22q11.2DS have 2.4 to 3.0 Mb of gene fragment deletion, containing more than 50 pathogenic genes, such as *TBX1*, *PROD*, and *PI4KA*.^14^ Among them, the deletion of TBX1 is an important factor causing congenital heart disease.^10–15,16^ In our case, the female patient did not have any special facial features, congenital palate abnormalities, immunodeficiency, learning difficulties, mental disorders or other abnormalities. IAA was discovered by cardiac ultrasound during her second pregnancy. We determined that she had a 2.35-Mb deletion in 22q11.21, which contained a single-dose sensitive gene *TBX1*. The patient’s cardiac abnormalities were consistent with the clinical phenotype of 22q11.2DS.

Studies have shown that 22q11.2DS in patients is approximately 50% likely to be inherited to the next generation through autosomal dominant inheritance. Approximately 8% of these patients are affected by parental inheritance.^13^ The common techniques for prenatal genetic diagnosis include chromosome karyotyping analysis, chromosomal microarray (CMA), copy number variation sequencing (CNV-seq), and WES. Chromosome karyotyping analysis is a study of chromosomes in the metaphase of cell division, and it can detect abnormal chromosome numbers and structural abnormalities >5 to 10 Mb. Chromosome karyotyping analysis is relatively simple and convenient for clinical use. However, chromosome karyotype analysis has difficulty in detecting chromosomal diseases such as microdeletions compared with other technologies. CMA is a high-resolution whole genome screening technology used to detect chromosomal structural abnormalities and gene copy number variations. CMA can check aneuploidy of the entire genome, structural abnormalities of large chromosomal fragments, and microdeletions and microduplications. However, CMA cannot detect chromosomal balanced translocations, gene level variations, and low chimerism rates.^17^ CNV-seq is a genome detection technique based on second-generation sequencing technology. CNV-seq has the advantages of high throughput and low cost compared with CMA. This technique can detect insertion or deletion variations in gene sequences ranging from 50 to 5 mb. CNV-seq can also detect chromosomal aneuploidy and copy number variations, but may not detect chromosomal aneuploidy, heterozygosity loss, and chimeric chromosomal abnormalities. Furthermore, this technique is unable to detect chromosomal balance structure rearrangements and gene point mutations. WES is a method used for detecting all exons of human genes. In contrast to CNV-seq and CMA, the detect resolution of WES can reach the level of identifying single base mutations.^18^ However, the reporting cycle of WES is relatively long. Clinical doctors have high requirements for interpretating WES because of the complexity of its results.^19^ In our case, the patient did not have other symptoms of 22q11.2DS, and there were no related patients in her family. We could not rule out the possibility of other genetic diseases besides 22q11.2DS. Therefore, we performed a WES examination. There can be clinical variability of 22q11.2DS, and the genetic probability of 22q11.2DS is 50%. Although the patient’s two children did not show any clinical manifestations related to 22q11.2DS, we suggested that her two children undergo genetic testing, but she refused these examinations.

In a study of 22q11.2DS, immunological evaluation of neonates at birth improved the detection rate of this disease.^20^ In addition, in a study of an immune system disease model, researchers found that patients with 22q11.2DS and thymus and T cell deletion were more frequently identified in children than adults.^21^ Therefore, the onset of 22q11.2DS may have different clinical manifestations according to age. Our patient did not have any previous abnormalities of immune function or thyroid function. IAA was not detected until the second pregnancy by a cardiac ultrasound examination, and WES confirmed the cause of the disease as 22q11.2DS. No abnormalities were found in thyroid ultrasound or thyroid function tests of this patient. We were unable to confirm the state of the patient’s T cells and unable to determine whether there was a thymus or immune deficiency in her childhood. This inability to detect immune and thyroid abnormalities may be one of the reasons why many patients do not have an early diagnosis.

## Conclusion

IAA is a type of malformation. The most common genetic finding associated with IAA is 22q11.2, and when this is suspected, genetic testing facilitates an etiological diagnosis and thus improves maternal and neonatal outcomes.

## Supplemental Material

sj-pdf-1-imr-10.1177_03000605241306905 - Supplemental material for Patient identified with interrupted aortic arch and 22q11.2 deletion syndrome in late pregnancy: a case report Supplemental material, sj-pdf-1-imr-10.1177_03000605241306905 for Patient identified with interrupted aortic arch and 22q11.2 deletion syndrome in late pregnancy: a case report by Kejun Pu, Mengya Yang, Meng Xia, Shuxin He, Hongbi Song and Jing Wu in Journal of International Medical Research

## Data Availability

Data sharing is not applicable to this article because no new data were created or analyzed in this study.
