# Supplementary material for: Patient identified with interrupted aortic arch and 22q11.2 deletion syndrome in late pregnancy: a case report
Source: J Int Med Res. 2024 Dec 29;52(12):03000605241306905. doi: 10.1177/03000605241306905 (PMC11686636; doi:10.1177/03000605241306905)
Supplement: sj-pdf-1-imr-10.1177_03000605241306905 - Supplemental material for Patient identified with interrupted aortic arch and 22q11.2 deletion syndrome in late pregnancy: a case report [file sj-pdf-1-imr-10.1177_03000605241306905.pdf]

手术知情同意书

姓名：[redacted] [redacted] 床号：[redacted]

医疗介绍和治疗建议：

医生已告知我目前诊断：，需要在手术日期进行腹膜内子宫下段横切口剖宫产术。

其他可供选择替代治疗方案：1、继续待产观察

2、经阴道分娩

3、其他医院治疗

我（患者近亲属、代理人）对上述推荐治疗方案及可选择替代治疗方案的优缺点和实施上述推荐治疗方案的必要性已充分了解，同意选择推荐治疗方案进行手术治疗。

手术潜在风险和对策：

医生告知我手术可能发生的一些风险，有些不常见的风险可能没有在此列出，具体的手术方式根据不同病人的情况有所不同，医生告诉我可与我的医生讨论有关我手术的具体内容，如果我有特殊的问题可以与我的医生讨论。

一、我理解任何手术麻醉都存在风险。

二、我理解任何所用药物都可能产生副作用，包括轻度的恶心、皮疹等症状到严重的过敏性休克，甚至危及生命。

三、我理解此手术可能发生的风险：

（一）术中：1.麻醉意外；2.心、脑血管意外；3.大出血；4.邻近脏器、血管、神经损伤；

（二）术后：1.切口感染或不愈合；2.迟发性大出血或血肿；3.血管栓塞；4.多器官衰竭；

（三）专科情况：

1.术中或术后诱发或加重潜在内科疾病，致心脑血管意外、呼吸衰竭、心力衰竭，血栓形成，严重危及生命甚至猝死。

2.羊水栓塞、DIC，严重危及生命。

3.子宫收缩乏力或胎盘粘连、植入等致大出血，危及生命，产后发生席汉氏综合征，必要时需行宫腔填塞纱条、子宫B-lynch缝合术，术后宫腔粘连，继发不孕，甚至切除子宫，丧失生育能力、无月经来潮等。

4.若术中探查发现他科情况，如卵巢囊肿、子宫肌瘤等，根据术中具体情况考虑剔除，可能会增加手术难度及出血风险，也有复发可能，必要时再次手术；若术中情况不允许同时处理，必要时长期随诊，再次手术可能，术后病检若提示恶性病变等，必要时再次手术或其他治疗。

5.术中损伤肠管、膀胱等，根据具体情况，必要时行修补或造瘘术，术后可能出现肠粘连、梗阻、肠瘘、腹膜炎等，必要时长期保留尿管、造瘘管，甚至可能再次手术。

6.胎儿宫内窘迫，窒息，窘死，新生儿损伤，吸入性肺炎、新生儿肺不张、新生儿缺血缺氧性脑病、新生儿窒息甚至死亡等，新生儿先天性畸形不能除外，术中取头困难，必要时需行产钳助产，可能造成新生儿头颅损伤，新生儿头皮血肿、新生儿颅内出血、新生儿臂丛神经损伤、锁骨神经损伤等，新生儿早产不除外，早产儿存活率低，死亡率高，各器官发育不成熟，新生儿产伤不除外。

7.术后各种感染，高热不退，坠积性肺炎，甚至败血症等危及生命，包括腹部切口感染、脂肪液化，切口不愈合或愈合不良，需要长期换药，必要时二期缝合。

8.术后肠粘连、肠梗阻，直肠膀胱功能障碍，慢性盆腔痛。

9.术后子宫内膜异位症、子宫切口憩室发生不除外。

10.术后大出血，必要时需行子宫动脉介入栓塞术，再次手术切除子宫等，丧失生育能力、无月经来潮。

11.其他医学上预测不到的情况。

12.术中可能使用高值耗材。

13.（1）术后严格避孕2-3年；（2）若行双侧输卵管结扎术，可能发生输卵管或输卵管系膜出血、血肿形成，输卵管瘘道形成、感染、局部粘连等导致异位妊娠，输卵管再通，引起再次妊娠甚至异位妊娠可能，术后如有再次妊娠的意愿，则行输卵管复通术可能效果不佳甚至难以再通等。

姓名：[redacted] [redacted] 床号：[redacted]

一旦出现上述情况，检查项目将增加，治疗时间将延长，可能再次或多次手术，从而产生相应的费用。

四、我理解如果我患有高血压、心脏病、糖尿病、肝肾功能不全、静脉血栓栓塞症等疾病或者有吸烟史，以上这些风险可能会加大，或者在术中或术后出现相关的病情加重或心脑血管意外，甚至死亡。

五、我理解术后如果我的体位不当或不遵医嘱等，可能影响手术效果、发生静脉血栓栓塞症等可能特殊风险或主要疾危因素。

患者知情选择：

- 我理解手术治疗之外的治疗方法，并理解若不行手术治疗可能导致的后果，经慎重考虑，选择手术治疗。
- 我的医生已经告知我将要进行的手术作方式、此次手术及手术后可能发生的并发症和风险、可能存在的其它治疗方法并且解答了我关于此次手术的相关问题。
- 我同意在手术中医生可以根据我的病情对预定的手术方式做出调整。
- 我理解我的手术可能需要多位医生共同进行。
- 我理解我的手术可能因特殊情况，需更换相应资质的医师；可能因病情复杂，需要其他专业的医师术中会诊，并可能共同协作完成手术。
- 我并未得到手术百分之百成功的许诺。
- 我授权医师对操作切除的病变器官、组织或标本进行处置，包括病理学检查、细胞学检查和医疗废物处理等。

医生陈述：　我已经告知患者将要进行的手术方式、此次手术及手术后可能发生的并发症和风险、可能存在的其它治疗方法并且解答了患者关于此次治疗的相关问题。

医生签名：[redacted]　　签名日期：[redacted]

患者签名：[redacted]　　签名日期：[redacted]

如果患者无法签署知情同意书，请其授权的近亲属/法定代理人在此签名：

患者授权近亲属/法定代理人签名：　　与患者关系：　　签名日期：
